# Staged Endovascular and Surgical Management of Pediatric Tracheo-Innominate Artery Fistula: A Case Report

**DOI:** 10.3400/avd.cr.25-00087

**Published:** 2026-02-17

**Authors:** Yuchen Cao, Masaaki Koide, Masafumi Yashima, Hisashi Sugiyama, Yasumi Nakashima, Arika Matsushita, Kotaro Ishida, Yusuke Okui

**Affiliations:** 1 Department of Cardiovascular Surgery, Seirei Hamamatsu General Hospital, Hamamatsu, Shizuoka, Japan; 2 Department of Pediatric Cardiology, Seirei Hamamatsu General Hospital, Hamamatsu, Shizuoka, Japan; 3 Department of Otorhinolaryngology, Seirei Hamamatsu General Hospital, Hamamatsu, Shizuoka, Japan; 4 Department of Anesthesiology, Seirei Hamamatsu General Hospital, Hamamatsu, Shizuoka, Japan

**Keywords:** endovascular stent graft, staged surgery, tracheo-innominate artery fistula

## Abstract

Tracheo-innominate artery fistula (TIF) is rare but potentially fatal, especially in pediatric patients. We present a case treated by emergency endovascular stenting followed by elective open surgery. Stent grafting achieved immediate hemostasis and served as a lifesaving bridge, but its limitations—including risks of infection, rebleeding, and graft mismatch due to somatic growth—made definitive surgery necessary. Laryngoscopic findings revealed intratracheal graft exposure, prompting timely graft removal and tracheal repair. This staged strategy highlights both the value of stenting as bridging therapy and the importance of early multidisciplinary planning in pediatric TIF.

## Introduction

Tracheo-innominate artery fistula (TIF) is a rare but potentially fatal complication of tracheostomy. The reported incidence among tracheostomized patients is 0.1%–1%. However, most cases are adults, with few reports of TIF in pediatric patients.^1,2)^ The standard treatment is emergency open surgery, such as innominate artery ligation or resection.^2)^ However, due to the high surgical risk and perioperative mortality, endovascular stent grafting has emerged as a less invasive alternative for rapid hemostasis.^1–4)^ Yet, while endovascular therapy may serve as an effective initial measure, subsequent surgical intervention is often required in cases with airway erosion or infection risk.^2,4)^ In pediatric patients, additional concerns include complications due to somatic growth, such as stent graft mismatch or migration.^3)^

This report describes a rare pediatric case of TIF successfully managed with initial endovascular stenting to control bleeding, followed by elective open surgery. Such preemptive surgical intervention has seldom been reported in children.

## Case Report

A 9-year-old boy with severe psychomotor retardation due to neonatal asphyxia and cerebral palsy presented 8 months after undergoing a tracheostomy for laryngotracheomalacia. The patient had previously undergone pulmonary artery banding and subsequent debanding for pulmonary hypertension associated with a double outlet right ventricle. He remained cyanotic on home oxygen (baseline oxygen saturation, 85%–90%).

**Fig. 1:**
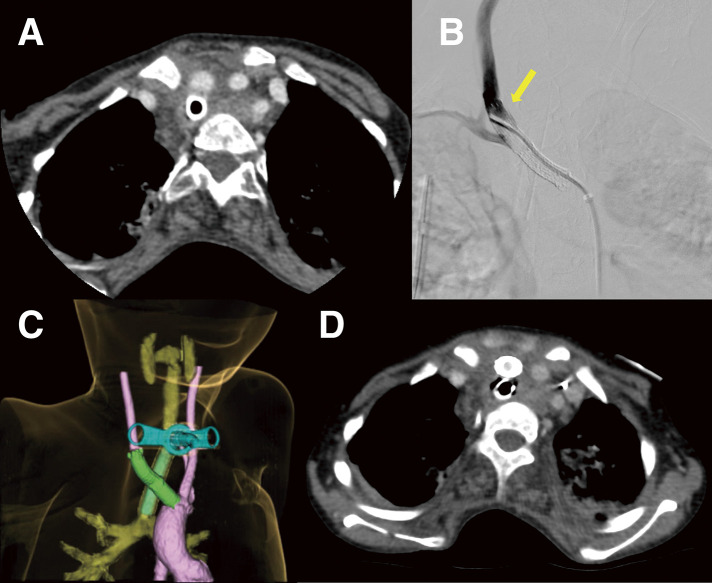
Representative imaging of a pediatric TIF from diagnosis to early postoperative assessment. (**A**) Axial contrast-enhanced computed tomography showed close apposition between the innominate artery and the trachea at the level of the tracheostomy tube cuff, without evidence of pseudoaneurysm formation or contrast extravasation. (**B**) Postoperative angiography at the distal edge of the VIABAHN stent graft (W. L. Gore & Associates, Flagstaff, AZ, USA) demonstrated a small type Ib endoleak (arrow). (**C**) Postoperative computed tomography on day 3 showed patent stent grafts without endoleaks. The proximal end of the VIABAHN stent graft extended slightly into the aortic arch. (**D**) An axial image demonstrated no contrast leakage between the stent graft and the native vessel wall, confirming complete exclusion of the fistula. TIF: tracheo-innominate artery fistula

While at school, the patient pulled on his tracheostomy tube, resulting in massive bleeding and desaturation. After temporary hemostasis, bleeding recurred during airway assessment at an outpatient otorhinolaryngology clinic. A laryngoscopy could not identify the source of the bleeding from the tracheostomy tube to the carina, raising suspicion of a TIF just above the cuff. Contrast-enhanced computed tomography (CT) showed contact between the innominate artery and the trachea at the tracheostomy cuff level. There was no contrast extravasation (**Fig. 1A**).

Considering the patient’s temporary hemodynamic stability and risks of infection and rebleeding, a multidisciplinary team initially planned surgical transection via partial sternotomy. Replacement of the tracheostomy with an oral endotracheal tube was deemed the best means of ventilation. Balloon occlusion of the innominate artery before tube exchange was considered; however, because balloon inflation could have imposed mechanical stress on the fragile fistula and potentially enlarged the defect, we opted to perform a simple, rapid tube exchange to minimize additional manipulation. In retrospect, temporary balloon occlusion might have reduced airway bleeding, but our priority at the time was to avoid further mechanical stress on the friable innominate artery.

In the operating theater, loosening of the tracheostomy tube fixation led to profuse airway bleeding, resulting in hemorrhagic shock. As the bleeding could not be controlled and open surgery was too risky, we decided on an emergency endovascular approach. After obtaining informed consent, off-label use of a stent graft (approval no. 4751) was later submitted to the ethics board. Left femoral arterial access was obtained via surgical cutdown at the operating surgeon’s discretion, and a 7-Fr sheath was advanced to the innominate artery. Although a transpercutaneous approach is commonly used, the patient’s small femoral artery and the emergent situation favored cutdown to ensure reliable access. A 7 × 25-mm VIABAHN stent graft (W. L. Gore & Associates, Flagstaff, AZ, USA) was deployed within the innominate artery to seal the fistula. Because the innominate artery was very short and only a 7 × 25- or an 8 × 50-mm device was available emergently, using the longer device would likely have crossed and obstructed the origin of the right subclavian artery. Although undersized, the shorter device enabled us to cover the fistula while preserving right subclavian artery flow during initial stabilization. However, we found a small type Ia endoleak. When the cuff was deflated, bleeding recurred. An additional 9 × 29-mm Omnilink Elite stent (Abbott Vascular, Santa Clara, CA, USA) was placed to increase radial force, but balloon dilation displaced the initial stent graft proximally by 5 mm, causing another major bleed (**Supplementary Video 1**). The only remaining graft available was an 8 × 50-mm VIABAHN stent graft. This was deployed overlapping the first and extended to the end in the right common carotid artery (CCA). Although a small type Ib endoleak persisted (**Fig. 1B**), hemostasis was achieved. The procedure was concluded when bleeding cessation continued after tracheostomy tube cuff deflation.

**Fig. 2:**
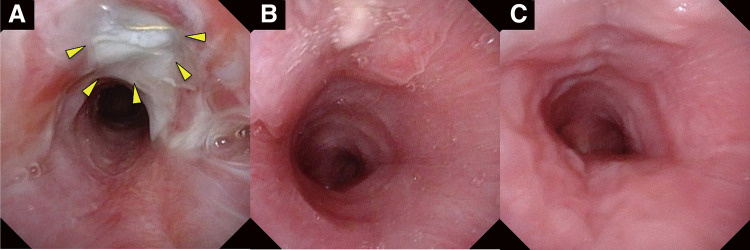
Laryngoscopic images from onset to mucosal recovery in a 9-year-old patient with tracheo-innominate artery fistula. (**A**) An initial laryngoscopy revealed exposure of the patient’s VIABAHN stent graft (W. L. Gore & Associates, Flagstaff, AZ, USA) within the tracheal lumen (arrows). (**B**) Follow-up laryngoscopies at 1 month and (**C**) 2 months showed progressive epithelial healing of the tracheal defect.

Postoperative intensive care stabilized the patient’s hemodynamics and improved his ventilation. CT on day 3 confirmed patent stent grafts without endoleak (**Fig. 1C**). The right CCA was opacified from the innominate artery, while the right subclavian artery was supplied via the vertebral artery during the arterial phase. Aspirin was started on day 13 to prevent thrombosis. On day 18, the patient exhibited left hemiparesis and right gaze deviation, although the onset may have been earlier, but difficult to recognize because of his severe psychomotor retardation. Contrast-enhanced CT performed on day 20, prompted by these neurological findings, revealed a large hemorrhagic infarction in the right middle cerebral artery region. The same CT demonstrated no thrombosis within the VIABAHN stent graft, indicating that graft occlusion was unlikely. The cause of the infarction remained uncertain, but global hypoperfusion due to hemorrhagic shock may have contributed, possibly further exacerbated by transient balloon occlusion of the innominate artery in an already compromised cerebral circulation. Laryngoscopy revealed exposure of the VIABAHN stent graft in the trachea (**Fig. 2A**). **Figure 2** presents a series of laryngoscopic images demonstrating the chronological course. A multidisciplinary conference planned definitive surgery on day 53 after the initial stent grafting, to avoid infection or rebleeding. The integrity of the Willis circle was confirmed by CT. No revascularization was planned due to a preexisting right hemorrhagic infarction.

**Fig. 3:**
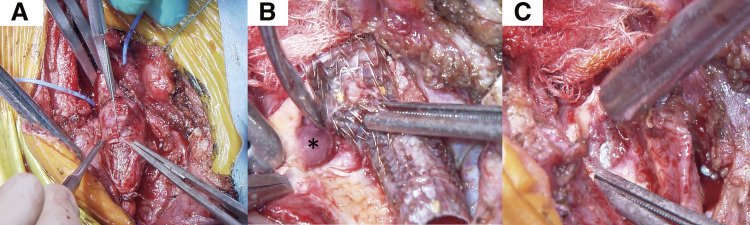
Intraoperative findings in a 9-year-old patient with a TIF. (**A**) After clamping of the proximal innominate artery and ligation of the right common carotid and subclavian arteries, a longitudinal arteriotomy from the right common carotid to the innominate artery was used to expose the patient’s VIABAHN stent graft (W. L. Gore & Associates, Flagstaff, AZ, USA). (**B**) The site of the TIF showed gelatinous, poorly vascularized granulation tissue (*), indicating severely impaired wound healing. (**C**) The tracheal cuff of the endotracheal tube was visible through the tracheal defect, confirming the location of the fistula. TIF: tracheo-innominate artery fistula

Under general anesthesia, access was gained via a partial sternotomy. A curved skin incision was made, and parts of the right clavicle and sternum were resected. Test clamping of the right CCA showed pressure of 40 mmHg and mean systemic pressure of 50 mmHg, suggesting good collateral flow. The right CCA and the subclavian artery were ligated, with no significant change in right upper extremity blood pressure. Near-infrared spectroscopy confirmed stable cerebral oxygenation. Revascularization was deemed unnecessary. The proximal portion of the innominate artery was clamped, including part of the VIABAHN stent graft. The artery was transected near the aortic arch. The proximal stump was closed with double-layer sutures and reinforced with mattress sutures. The stent graft was removed via a longitudinal incision from the right CCA to the innominate artery. The fistula was closed using double-layer suturing of the healthy arterial wall. The space around the closure was adequately filled with thymic and sternocleidomastoid tissue, so no pectoralis major flap was required (**Fig. 3** and **Supplementary Video 2**).

Postoperatively, no new neurological deficits manifested. Follow-up laryngoscopies at 1 and 2 months confirmed good healing (**Fig. 2B** and **2C**). The patient was discharged on postoperative day 165.

## Discussion

In this case, the patient exhibited no signs of infection or recurrent bleeding. However, early laryngoscopic findings of intraluminal stent graft exposure prompted definitive surgical intervention. We interpreted this as a potential pre-infective and pre-penetrative state, with a substantial risk of catastrophic complications. Elective surgical repair at this early stage enabled safe graft removal and tracheal reconstruction, preventing infection and rebleeding. The timing of definitive surgery was determined after confirming persistent tracheoscopic findings with no evidence of healing and ensuring neurological stability and adequate cerebral perfusion through multidisciplinary evaluation. The procedure was performed approximately 7 weeks after stent grafting, consistent with previous reports describing second-stage surgery several weeks to months after initial endovascular treatment, depending on recovery and airway status.^5,6)^ In pediatric patients, this strategy mitigates the long-term risks associated with indwelling stent grafts, such as mismatch or migration due to somatic growth.

The patient’s stent graft became exposed within the tracheal lumen without evidence of healthy granulation tissue formation. Normally, epithelialization proceeds over granulation tissue during the healing process,^2)^ but only a small amount of friable tissue was observed, suggesting impaired wound healing and possible latent infection in the contaminated airway. Such delayed healing has been reported to predispose to infection or further tracheal erosion, emphasizing that endovascular repair should serve only as a temporizing measure until definitive surgery can be performed.^4,7)^ Serial postoperative laryngoscopy confirmed persistent graft exposure with no epithelialization, reinforcing the concern that the wound was unlikely to heal spontaneously and that serious complications could develop. Although reports explicitly describing proactive surgical intervention based solely on endoscopic findings are scarce, the concept is consistent with previous recommendations that early surgery should be considered when healing does not progress or when signs of infection or erosion emerge. Our case underscores the importance of recognizing impaired wound healing as an early indication for definitive surgery in selected patients.

To achieve rapid hemodynamic stabilization and serve as a bridge to definitive surgery, initial endovascular intervention was deemed necessary. Early definitive repair is desirable; however, this patient developed hemodynamic collapse upon operating room entry, making immediate open repair unsafe. Emergency endovascular stenting was therefore performed as a life-saving measure, serving as a bridge to definitive surgery. Although endovascular therapy is increasingly utilized, it is often associated with complications such as infection, graft migration, or rebleeding, particularly in cases with airway erosion or incomplete healing.^2,3)^ In pediatric patients, there are also risks of progressive graft mismatch, positional shift, or structural failure over time due to somatic growth.^3)^ These limitations reinforce the importance of endovascular stenting as a temporizing, rather than definitive, solution in this population.

The patient subsequently developed a hemorrhagic cerebral infarction. Contrast-enhanced CT on day 20 revealed a large infarction in the right middle cerebral artery region but no thrombosis within the VIABAHN stent graft, making graft occlusion unlikely. The cause was uncertain, but global hypoperfusion from hemorrhagic shock may have contributed. Post-traumatic vasospasm and cerebral hypoperfusion have been documented in patients with traumatic brain injury on serial CT angiography and perfusion imaging.^8)^ Pediatric patients may be especially vulnerable to delayed ischemia due to autoregulatory instability. Severe vasospasm in childhood has been associated with arterial ischemic stroke in case reports.^9)^ Even a brief balloon occlusion of the innominate artery could have further compromised flow in an already low-perfusion state. Although no residual endoleak was detected on postoperative CT, this case highlights the importance of early correction of even minimal endoleak, postoperative assessment of cerebral perfusion, and individualized antithrombotic management to minimize ischemic risk in future cases.

TIFs in pediatric patients present unique challenges due to the limited availability of appropriate device sizes and considerations such as the short length and variable diameter of the innominate artery. The emergency use of available in-house devices likely explains the initial undersizing of the stent graft in our patient, which resulted in migration and secondary bleeding. This case highlights the importance of meticulous planning and careful device selection, particularly in pediatric patients.

## Conclusion

We report a rare pediatric case of TIF managed with emergency endovascular stent grafting followed by elective open surgery. Although the surgical outcome was favorable, this experience highlights important lessons regarding the limitations of emergency stent selection and the potential for delayed complications such as hemorrhagic infarction. Based on early endoscopic findings, proactive definitive surgery prevented infection and rebleeding. This case underscores the importance of continuous airway surveillance, timely decision-making, and multidisciplinary planning to optimize outcomes in future pediatric TIF cases.

## Supplementary Materials

Supplementary Video 1 Balloon expansion of an initially undersized stent graft leading to proximal migration and airway rebleeding. Dilation of a balloon-expandable Omnilink Elite stent (Abbott Vascular) placed within the initially undersized 7 × 25-mm VIABAHN stent graft (W. L. Gore & Associates) was performed using a 10 × 20-mm Mustang balloon (Boston Scientific, Natick, MA, USA). The larger balloon was selected to achieve rapid hemostasis in an emergency, although this exceeded the nominal stent diameter. During inflation, the VIABAHN stent graft migrated proximally by approximately 5 mm, leading to the recurrence of massive airway bleeding.

Supplementary Video 2 Intraoperative video of stent graft removal and tracheal fistula repair via partial sternotomy in a 9-year-old patient with a tracheo-innominate artery fistula. The innominate artery was transected, and the graft was removed. Friable granulation tissue was found at the fistula site, and the tracheal cuff was visible through the lumen. After thorough irrigation, the artery was closed *in situ*, and the defect was covered using the thymus and the sternocleidomastoid muscle without a pectoralis major flap.
